# IMPROVING PRIMARY CARE PROVIDER EDUCATION AND SATISFACTION VIA THE CATCH-ON LEARNING COMMUNITIES

**DOI:** 10.1093/geroni/igz038.140

**Published:** 2019-11-08

**Authors:** Erin Emery-Tiburcio, Magdalena Bednarcyzk, Febe Wallace, Michelle Newman

**Affiliations:** 1 Rush University Medical Center, Chicago, Illinois, United States; 2 Cherokee Health Systems, Morristown, Tennessee, United States

## Abstract

Nationally, there is a shortage of geriatric trained healthcare providers caring for older adults. As the population of older adults grows, health care systems and primary care providers struggle to provide high quality, cost effective care for older adults. Time for training is also limited in busy community health centers. The CATCH-ON Learning Communities (LCs) are telehealth educational interventions based on the ECHO model, modified to be less time intensive, thus decreasing cost to participating clinics. In the LC, geriatric specialists provide evidence-based, best practice training utilizing case discussions to illustrate pertinent learning points via monthly one hour video conferences. Practical, specific behavioral recommendations are offered for immediate implementation in each session. LCs are provided to interprofessional primary care teams. The first LC with a federally-qualified health center (FQHC) yielded consistently high satisfaction from participants, along with a 17% decrease in high risk medication prescriptions and 22% increase in falls screenings. Training the primary care workforce in evidence based geriatric interventions can improve the care of all older adults within each health system, improving healthcare access to help mitigate healthcare inequalities, slow adoption of best practices and rising costs of caring for complex older adults. The CATCH-ON Learning Community is an effective, low cost model of training the primary care work force without geographical or financial constraints that frequently limit access to specialized care.

